# Mitochondrial genome of Chinese grass shrimp, *Palaemonetes sinensis* and comparison with other Palaemoninae species

**DOI:** 10.1038/s41598-019-53539-x

**Published:** 2019-11-21

**Authors:** Yingying Zhao, Xiaochen Zhu, Yingdong Li, Zhibin Han, Weibin Xu, Jing Dong, Hua Wei, Xiaodong Li

**Affiliations:** 1 Key Laboratory of Zoonosis of Liaoning Province, College of Animal Science and Veterinary Medicine, Shenyang Agricultural University, Shenyang, 110866 China; 2 Panjin Guanghe Crab Industry Co.Ltd., Panjin, 124000 China

**Keywords:** Agricultural genetics, Mitochondrial genome

## Abstract

The mitogenome of Chinese grass shrimp, *Palaemonetes sinensis*, was determined through Illumina sequencing, and the basic characteristics and gene arrangement were analyzed. The mitogenome of *P*. *sinensis* was 15955 bp in length, consisting of 13 protein-coding genes (PCGs), 22 *tRNA* genes, 2 *rRNA* genes and one control region, with tightly packed. 33 of these genes were encoded on the heavy strand, and the remainders encoded on the light strand. The composition of *P*. *sinensis* mitogenome presented a strong A + T bias, which account for 66.7%. All PCGs were initiated by a canonical ATN codon, except *nad5*, which was initiated by GTG. The termination codons of the PCGs were TAA, TAG and T–. The secondary structures of 22 *tRNAs* of *P*. *sinensis* had the typical clover structure, except of *trnS1* owing to the lack of dihydroxyuridine (DHU) arm. Gene order comparison of *P*. *sinensis* and previously-sequenced Palaemoninae revealed a unique translocation between *trnT* and *trnP* in Macrobrachium. The phylogenetic analyses showed that three Exopalaemon species formed a monophyletic group and then clustered with two Palaemon species and *P*. *sinensis* successively whereas Macrobrachium clustered with *Palaemon capensis* in the other clade.

## Introduction

Palaemonidae, as the second most species-rich family in Caridean, including 134 genera and 934 extant species^1^, is widely distributed in almost any aquatic habitat. Palaemonidae are divided into two subfamilies: Pontoniinae Kingsley, 1879 (108 genera, 562 species) and Palaemoninae Rafinesque, 1815 (26 genera, 372 extant species). Despite of the numerical dominance of Pontoniinae, researchers have done more works on Palaemoninae due to its wide distribution, economic value and ecological importance. Nevertheless, the phylogenetic relationship within this subfamily is still disputed because the current classification system failed to describe their underlying evolutionary relationship^2–4^. For example, Pereira pointed out the paraphyly on generic level based on his cladistic analysis of morphological characteristics^3^. Murphy & Austin found species belonging to three different genera, *Macrobrachium intermedium*, *Palaemon serenus*, and *Palaemonetes australis* formed a monophyletic assemblage instead of with their congeneric species^4^. The topology given by Cuesta *et al*. showed that species from Palaemon and Palaemonetes clustered according to global geographical distribution and by genera with the exception of the Australian Palaemonid shrimps, which demonstrated the dichotomy between Palaemon and Palaemonetes genera (absence/presence of the mandibular palp) was phylogenetically questionable^5^. Ashelby *et al*. suggested the reevaluation of morphological traits to separate the genus of Palaemon, Palaemonetes, Exopalaemon and Coutierella because some species from those genera present monophyly^6^. However, most molecular studies on Palaemoninae are based on the analysis of partial sequences of 16S rRNA and fragment of the nuclear genes histone3 (H3)^6,7^. Certainly, analysis of other gene sequences (both from mitogenome and nuclear genome) are necessary to improve the understanding of phylogenetic relationship amongst Palaemonid shrimps.

Animal mitochondrial DNAs are typically circular molecules, approximately between 14 and 18 kb in length, normally containing 13 protein-coding genes (PCGs), two ribosomal RNA genes (*rrnL* & *rrnS*), 22 transfer RNA (*tRNA*) genes, and one control region (CR)^8,9^. It has been widely accepted that mitogenome has rapid evolutionary rate and lack of genetic recombination^8^.

It had becoming increasingly popular to employ entire mitogenomes for phylogenetic relationship analyses^10–12^, which was due to the following reasons. Firstly, complete mitogenomes often reveal more genetic information because single genes or partial DNA sequences are often too short to provide adequate phylogenetic information^13^. Secondly, combination of mitochondrial and nuclear genomes makes model selection diffcult^14^, and the addition of rRNA makes alignment ambiguous^15^, Thirdly, some genome-level characters which are significantly important for phylogeny, such as gene order rearrangement, must be detected by comparison of entire mitogenomes^16–18^. Lastly, NGS make the complete mitogenome acquirement economically and not as time consuming as before^19^. So far ten mitogenome of Subfamily Palaemoninae which belonged to three genera (Palaemon, Exopalaemon and Macrobrachium) were determined, whereas none of Palaemonetes has been reported.

Chinese grass shrimp, *Palaemonetes sinensis* (Sollaud, 1911), is one of the important species of Palaemoninae, and widely distributed in China, Myanmar, Vietnam, Japan, southeastern Siberia and Sakhalin, with crucial ecological value and a certain degree of ornamental and economical value^20–22^. Except mitochondrial 16S rDNA and nuclear Histone (H3) gene sequences^5,6^, there was no report about mitogenome of *P*. *sinensis*. In this study, the complete mitogenome of *P*. *sinensis* was obtained through NGS. As the first mitogenome of Palaemonetes, it would contribute to a better understanding of phylogenetic relationship within Palaemoninae, particularly among Palaemonetes and other genera mentioned above. Additionally, it is of great importance for future study of genetic biodiversity of *P*. *sinensis*.

## Results and Discussion

### Genome composition

**Figure 1:**
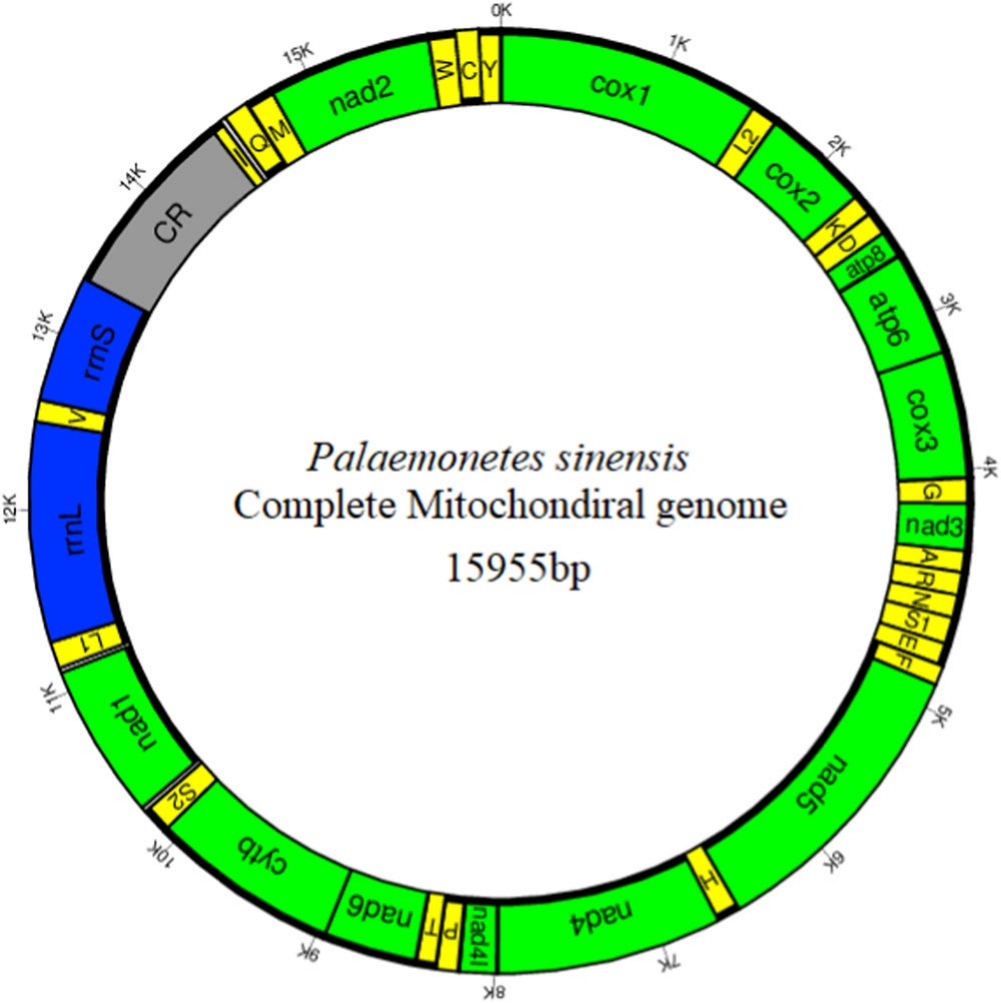
Graphical map of the mitogenome of *P*. *sinensis*. PCGs and ribosomal RNA genes are shown using standard abbreviations. Genes for transfer RNAs are abbreviated using a single letter. S1 = AGN, S2 = UCN, L1 = CUN, L2 = UUR. CR = control region. PCGs are green, tRNAs are yellow, rRNAs are blue, and CR is grey. Outside line and inside line indicate heavy strand and light strand, respectively. Bold line represents transcribed strand.

Approximate 5.2 million clean reads were obtained from raw sequences data and were clustered into 4,169,064 high quality reads. After further assembly, a circular mitogenome of 15,955 bp in length was finally generated (Figure 1). Compared with the other Palaemoninae species, *P*. *sinensis* mitogenome was slightly smaller than that of *Palaemon serenus* (15,967 bp), but it is in the range of the known Palaemoninae mitogenomes (15,694–15,967 bp). Nucleotide BLAST (blastn) of the entire mitogenome between *P*. *sinensis* and closely related species presented high similarity (77% with *Exopalaemon annandalei*, 76% with *Palaemon gravieri* and *Exo*p*alaemon modestus*).

The gene content of *P*. *sinensis* mitogenome was same as that of all known Palaemoninae, including 13 PCGs, 2 rRNA genes, and 22 *tRNA* genes plus a putative control region (Table 1 and Figure 1). Quite similar with Exopalaemon^10,23^ and Palaemon^24–26^, 23 of the 37 genes were coded on the H strand whereas the remaining 14 genes were transcribed on the L strand. Like most of Caridea, the mitogenomes of *P*. *sinensis* in this study were closely aligned, with only a small number of base overlapping between adjacent genes, indicating that RNA transcription and protein translation were more efficient (Table 1).

**Table 1:** Annotation of *P*. *sinensis* mitogenome.

Gene	Location	Gene length/bp	Start codon	Stop codon	Antic codon	H/L strand	Intergenic region length/bp
*cox1*	1–1542	1542	ATA	TAA		+	
*trnL2*	1543–1605	63			TAA	+	2
*cox2*	1608–2297	675	ATG	TAA		+	1
*trnK*	2299–2367	69			TTT	+	2
*trnD*	2370–2434	65			GTC	+	
*atp8*	2435–2593	159	ATG	TAG		+	-7
*atp6*	2587–3261	663	ATG	TAA		+	−1
*cox3*	3261–4049	783	ATG	TAA		+	6
*trnG*	4056–4120	65			TCC	+	
*nad3*	4121–4474	345	ATC	TAA		+	
*trnA*	4475–4535	61			TCG	+	−1
*trnR*	4535–4598	64			GTG	+	
*trnN*	4599–4663	65			GCT	+	
*trnS1*	4664–4730	67			TAG	+	
*trnE*	4731–4798	68			TTC	+	
*trnF*	4797–4860	64			GAA	−	−1
*nad5*	4860–6584	1725	GTG	TAA		−	
*trnH*	6585–6648	64			TAA	−	
*nad4*	6649–7983	1335	ATG	TAG		−	−7
*nad4l*	7977–8276	264	ATG	TAA		−	9
*trnP*	8286–8351	66			TGG	−	5
*trnT*	8357–8420	64			TGT	+	8
*nad6*	8428–8952	525	ATT	TAA		+	−1
*cyt b*	8952–10086	1134	ATG	T−		+	
*trnS2*	10087–10154	68			TGA	+	27
*nad1*	10182–11123	942	ATG	TAG		−	27
*trnL1*	11151–11216	66			GAA	−	
*rrnL*	11217–12514	1298				−	
*trnV*	12515–12579	65			TAC	−	−1
*rrnS*	12579–13368	790				−	
CR	13369–14527	1159					
*trnI*	14528–14594	67			GAT	+	32
*trnQ*	14627–14694	68			TTG	−	5
*trnM*	14700–14764	65			CAT	+	
*nad2*	14765–15760	942	ATG	TAG		+	−2
*trnW*	15759–15824	66			TCA	+	−1
*trnC*	15824–15886	63			GCA	−	
*trnY*	15887–15951	65			GTA−	−	11

The genome composition (A: 36.2%, G: 12.1%, T: 30.5%, C: 21.3%) presented a strong A + T bias, which account for 66.7% of the bases, and showed a AT skew ([A − T]/[A + T] = 0.085) and negative GC skew ([G − C]/[G + C] = −0.275). The AT skew was similar with *E*. *annandalei* (0.086) and higher than that of Palaemon and Exopalaemon (−0.049 in *E*. *modestus* to 0.057 in *Exopalaemon carinicauda*), but lower than that of Macrobrachium (0.100 in *M*. *lanchesteri* to 0.157 in *M*. *bullatum*) (Table 2). The GC skew of *P*. *sinensis* was similar with most of other previously sequenced Palaemoninae mitogenomes (Table 2). However, different regions of mitogenome had different A + T contents. The CR had the highest A + T content (84.8%), whereas the PCG region had the lowest A + T content (63.7%) (Table 3).

**Table 2:** Genomic characteristics of Palaemoninae mitogenome acquired from GenBank.

Species	Accession No.	Size (bp)	Nucleotide composition/%					AT-skew	GC-skew
			A	G	T (U)	C	A + T (U)		
*Palaemonetes sinensis*	MH880828	15,955	36.2	12.1	30.5	21.3	66.7	0.085	−0.275
*Palaemon serenus*	KM978916.1	15,967	29.2	16.9	29.8	24.1	59	−0.010	−0.176
*Palaemon gravieri*	KT935323.1	15,735	35.3	12.1	32.1	20.4	67.4	0.047	−0.255
*Palaemon gravieri*	KU899135.1	15,740	35.3	12.1	32.1	20.5	67.4	0.047	−0.258
*Palaemon capensis*	MF797833.1	15,925	36.2	11.5	32.9	19.4	69.1	0.048	−0.256
*Exopalaemon annandalei*	MG787410.1	15,718	34.8	12.7	29.3	23.2	64.1	0.086	−0.292
*Exopalaemon modestus*	MF687349.1	15,736	32.1	20.4	35.4	12.1	67.5	−0.049	0.255
*Exopalaemon carinicauda*	EF560650.1	15,730	33.6	13.4	30.0	23.0	63.6	0.057	−0.264
*Macrobrachium bullatum*	KM978918.1	15,774	37.3	11.7	27.2	23.7	64.5	0.157	−0.339
*Macrobrachium lanchesteri*	FJ797435.1	15,694	36.9	12.1	30.2	20.8	67.1	0.100	−0.264
*Macrobrachium nipponense*	HQ830201.1	15,806	37.2	12.5	28.9	21.5	66.1	0.126	−0.265
*Macrobrachium rosenbergii*	AY659990.1	15,772	35.8	13.4	26.4	24.3	62.2	0.151	−0.289

**Table 3:** Composition and skewness in PCGs, tRNAs, rRNAs, and CR Region of different Palaemoninae mitogenomes.

Species	Size (bp)	Nucleotide composition/%					AT-skew	GC-skew
		A	G	T (U)	C	A + T (U)		
**PCGs**								
*P*. *sinensis*	11166	34.8	12.9	28.9	23.4	63.7	0.093	−0.289
*P*. *serenus*	11076	21.7	21.3	34.3	22.7	56.0	−0.225	−0.032
*P*. *capensis*	11128	27.8	16.4	39.0	16.8	66.8	−0.168	−0.012
*P*. *gravieri*	11125	27.1	17.0	38.1	17.8	65.2	−0.169	−0.023
*P*. *gravieri*	11128	27.2	17.0	38.0	17.8	65.2	−0.166	−0.023
*E*. *annandalei*	11126	25.3	18.3	36.2	20.2	61.5	−0.177	−0.049
*E*. *modestus*	11136	27.2	16.9	37.9	18.0	65.1	−0.164	−0.032
*E*. *carinicauda*	11122	25.0	18.8	35.6	20.7	60.6	−0.175	−0.048
*M*. *bullatum*	11126	26.7	17.6	35.4	20.3	62.1	−0.140	−0.071
*M*. *lanchesteri*	11125	27.7	16.5	37.5	18.3	65.2	−0.150	−0.052
*M*. *nipponense*	11128	27.6	16.8	36.7	18.9	64.3	−0.142	−0.059
*M*. *rosenbergii*	11126	25.9	18.3	34.2	21.5	60.1	−0.138	−0.080
**tRNA**								
*P*. *sinensis*	1438	34.4	15.2	30.4	20.0	64.8	0.062	−0.136
*P*. *serenus*	1449	31.3	17.6	30.5	20.6	61.8	0.013	−0.079
*P*. *capensis*	1438	35.4	12.9	34.8	16.9	70.2	0.009	−0.134
*P*. *gravieri*	1444	35.1	14.6	32.1	18.2	67.2	0.045	−0.110
*P*. *gravieri*	1444	35.2	14.6	32.0	18.2	67.2	0.048	−0.110
*E*. *annandalei*	1450	33.7	15.9	30.3	20.1	64.0	0.053	−0.117
*E*. *modestus*	1443	32.4	18.5	34.6	14.5	67.0	−0.033	0.121
*E*. *carinicauda*	1445	33.6	15.2	32.2	19.0	65.8	0.021	−0.111
*M*. *bullatum*	1443	35.6	14.3	29.8	20.3	65.4	0.089	−0.173
*M*. *lanchesteri*	1449	34.9	14.4	31.5	19.3	66.4	0.051	−0.145
*M*. *nipponense*	1450	35.0	14.8	30.7	19.4	65.7	0.065	−0.135
*M*. *rosenbergii*	1449	34.6	15.1	30.1	20.2	64.7	0.070	−0.144
**rRNA**								
*P*. *sinensis*	2088	38.9	9.1	34.6	17.4	73.5	0.059	−0.313
*P*. *serenus*	2176	32.4	14.5	33.5	19.5	65.9	−0.017	−0.147
*P*. *capensis*	2112	37.7	9.3	36.5	16.5	74.2	0.016	−0.279
*P*. *gravieri*	2092	38.5	9.6	34.4	17.5	72.9	0.056	−0.292
*P*. *gravieri*	2091	38.5	9.5	34.5	17.5	73.0	0.055	−0.296
*E*. *annandalei*	2088	38.2	10.0	32.7	19.2	70.9	0.078	−0.315
*E*. *modestus*	2128	34.8	16.9	38.3	10.0	73.1	−0.048	0.257
*E*. *carinicauda*	2142	38.0	10.6	33.6	17.7	71.6	0.061	−0.251
*M*. *bullatum*	2169	39.1	9.9	29.8	21.3	68.9	0.135	−0.365
*M*. *lanchesteri*	2154	39.4	9.7	31.7	19.3	71.1	0.108	−0.331
*M*. *nipponense*	2157	38.9	10.4	29.9	20.7	68.8	0.131	−0.331
*M*. *rosenbergii*	2157	38.3	11.1	27.7	22.9	66.0	0.161	−0.347
**CR**								
*P*. *sinensis*	1159	46.7	6.5	38.1	8.7	84.8	0.101	−0.145
*P*. *serenus*	1150	37.5	12.5	35.5	14.5	73.0	0.027	−0.074
*P*. *capensis*	1085	42.3	9.4	38.1	10.2	80.4	0.052	−0.041
*P*. *gravieri*	948	42.1	8.0	39.7	10.2	81.8	0.029	−0.121
*P*. *gravieri*	947	42.0	8.1	39.6	10.2	81.6	0.029	−0.115
*E*. *annandalei*	934	43.0	7.7	37.2	12.1	80.2	0.072	−0.222
*E*. *modestus*	952	38.3	10.1	43.7	7.9	82.0	−0.066	0.122
*E*. *carinicauda*	886	41.0	9.3	38.7	11.1	79.7	0.029	−0.088
*M*. *bullatum*	1002	42.8	7.3	39.1	10.8	81.9	0.045	−0.193
*M*. *lanchesteri*	861	41.7	7.3	41.0	10.0	82.7	0.008	−0.156
*M*. *nipponense*	950	42.4	9.2	37.5	10.9	79.9	0.061	−0.085
*M*. *rosenbergii*	931	39.5	9.6	36.2	14.7	75.7	0.044	−0.210

### Protein-coding genes

The PCG region formed 69.98% of the *P*. *sinensis* mitogenome, and was 11,166 bp in length totally. Among the 13 PCGs, nine genes (*cox1*, *cox2*, *atp8*, *atp6*, *cox3*, *nad3*, *nad6*, *cyt b* and *nad2*) were coded on H strand, while the rest four genes (*nad5*, *nad4*, *nad4l* and *nad1*) were on L strand. The 13 PCGs ranged in size from 159 to 1725bp (Table 2). Each PCG was initiated by a canonical ATN codon, except *nad5* which was initiated by a GTG codon. The termination codons of the PCGs were TAA, TAG and T. Eight of 13 PCGs, *cox1*, *cox2*, *atp6*, *cox3*, *nad3*, *nad5*, *nad4l* and *nad6* used a typical TAA termination codon, as well as *atp8*, *nad4*, *nad1* and *nad2* terminated with TAG, but *cyt b* had an incomplete termination codon, a single T (Table 1).

The number of bases in the 13 PCGs was A > T > C > G, and the A + T content of 13 PCGs was 63.7%, showed a strong A + T bias (Table 3), as well as a strong A and C bias, with the AT-skew GC-skew was 0.093 and −0.289, respectively. The slightly positive value of AT-skew for *P*. *sinensis* indicated a higher occurrence of A compared to T nucleotides, whereas that of the other mitogenomes were all negative. In addition, GC-skew value for *P*. *sinensis* was the biggest negative comparing to that of other mitogenomes (−0.012 to −0.080). With the exception of *P*. *sinensis*, species of Macrobrachium showed slight smaller negative AT-skew (−0.138 to −0.150) and bigger negative GC-skew (−0.052 to −0.080) (Table 3).

The average frequency of the protein-coding genes codon and was calculated and shown in Table 4. The preference codon (most frequently used to encode same amino acid) was shown in bold font and their RSCU of them were all greater than 1. RSCU was an important index to reflect the preference degree of codon usage intuitively^27^. The results of this study showed that the codons of all protein-coding genes had strong preference, and most RSCU of NNU and NNA (i.e. the codon with the third site U or A) were greater than 1, with higher frequency of usage. And this result was consistent with the result of *E*. *carinicauda*^10^.

**Table 4:** The codon number and relative synonymous codon usage in *P*. *sinensis* mitochondrial protein coding genes.

Codon	Count	RSCU	Codon	Count	RSCU	Codon	Count	RSCU	Codon	Count	RSCU
**UUU** (**F**)	**251**	**1**.**63**	**UCU** (**S**)	**116**	**2**.**68**	**UAU** (**Y**)	**74**	**1**.**21**	**UGU** (**C**)	**34**	**1**.**36**
UUC (F)	57	0.37	UCC (S)	20	0.46	UAC (Y)	48	0.79	UGC (C)	16	0.64
**UUA** (**L**)	**250**	**2**.**46**	UCA (S)	55	1.27	**UAA** (*)	**8**	**1**.**33**	**UGA** (**W**)	**77**	**1**.**5**
UUG (L)	80	0.79	UCG (S)	5	0.12	UAG (*)	4	0.67	UGG (W)	26	0.5
**CUU** (**L**)	**123**	**1**.**21**	**CCU** (**P**)	**52**	**1**.**4**	CAU (H)	31	0.81	CGU (R)	11	0.7
CUC (L)	33	0.32	CCC (P)	50	1.34	**CAC** (**H**)	**46**	**1**.**19**	CGC (R)	7	0.44
CUA (L)	99	0.97	CCA (P)	41	1.1	**CAA** (**Q**)	**55**	**1**.**49**	**CGA** (**R**)	**37**	**2**.**35**
CUG (L)	25	0.25	CCG (P)	6	0.16	CAG (Q)	19	0.51	CGG (R)	8	0.51
**AUU** (**I**)	**218**	**1**.**53**	**ACU** (**T**)	**96**	**1**.**84**	**AAU** (**N**)	**69**	**1**.**11**	AGU (S)	36	0.83
AUC (I)	67	0.47	ACC (T)	44	0.84	AAC (N)	55	0.89	AGC (S)	22	0.51
**AUA** (**M**)	**139**	**1**.**51**	ACA (T)	61	1.17	**AAA** (**K**)	**69**	**1**.**6**	**AGA** (**S**)	**61**	**1**.**41**
AUG (M)	45	0.49	ACG (T)	8	0.15	AAG (K)	17	0.4	AGG (S)	31	0.72
**GUU** (**V**)	**113**	**1**.**74**	**GCU** (**A**)	**108**	**1**.**7**	**GAU** (**D**)	**42**	**1**.**25**	GGU (G)	38	0.59
GUC (V)	21	0.32	GCC (A)	73	1.15	GAC (D)	25	0.75	GGC (G)	43	0.67
GUA (V)	92	1.42	GCA (A)	57	0.9	**GAA** (**E**)	**51**	**1**.**23**	**GGA** (**G**)	**102**	**1**.**59**
GUG (V)	34	0.52	GCG (A)	16	0.25	GAG (E)	32	0.77	GGG (G)	73	1.14

### Transfer RNAs, ribosomal RNAs, and CR region

**Figure 2:**
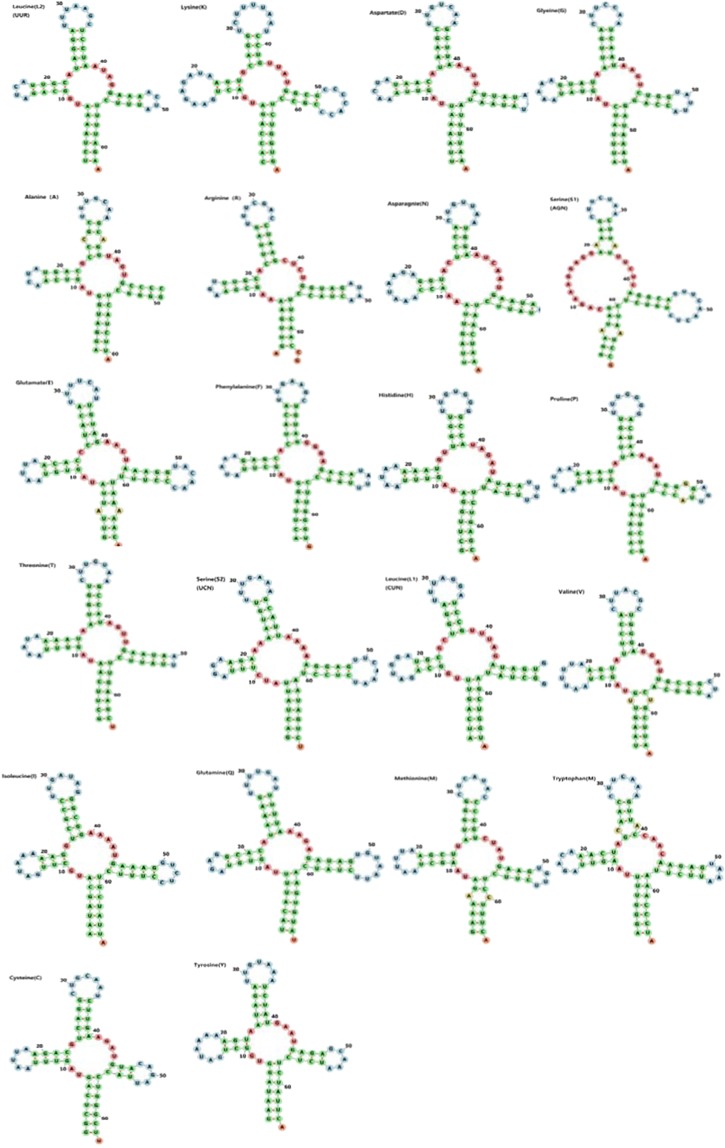
Predicted secondary structures of the 22 tRNA genes of the *P*. *sinensis* mitogenome.

Same to most Palaemoninae, *P*. *sinensis* mitogenome contained a set of 22 *tRNA*s genes (Figure 1). The *tRNA*s sequences ranged between 63 and 69 bp and exhibited a strong A + T bias (64.8%). Furthermore, they showed a positive AT skew (0.062) (Table 3). Fourteen *tRNA* genes were present on the H strand and eight were on the L strand. The secondary cloverleaf structure of 15 *tRNA*s was examined using tRNAscan-SE^28^, while the secondary cloverleaf structure of all the 22 *tRNA*s could examined using MITOS^29^. As a result, all the *tRNA* genes had the typical cloverleaf structure, except the *trnS1* gene, whose dihydroxyuridine (DHU) arm was replaced by a simple loop (Figure 2), which is a common feature in most Palaemonidae mitogenomes^10^.

The *rrnL* and *rrnS* genes were located between *trnL1* and *trnV* and between *trnV* and CR, respectively. The *rrnL* was 1298 bp, while *rrnS* was 790 bp in length. The CR was 1159 bp, and situated between *rrnS* and *trnI*. This region contains 84.8% A + T content, and had a positive AT skew (0.101) and negative GC skew (−0.145) (Table 3).

Compared with other Palaemoninae^10,23–26^, CR of *P*. *sinensis* mitogenome had different size. That was a common phenomenon, because it was generally believed that the length of control region has the largest variation of mitogenome^16^. *P*. *sinensis* had the highest composition of A nucleotides, and the lowest composition of G nucleotides, as well as the highest AT-skew value (Table 3).

### Gene arrangement

Among all known Palaemoninae sequences, gene order and orientation of the complete mitogenome of *P*. *sinensis* were identical to some previously-sequenced Palaemoninae, including three species of Palaemon (*P*. *serenus*, *P*. *gravieri* and *P*. *capensis*) and three species of Exopalaemon (*E*. *annandalei*, *E*. *modestus* and *E*. *carinicauda*) with the gene order was 5′-*nad4L*- *trnP*- *trnT* -*nad6*-3′)^10,23–26^. However, a rearrangement of translocation between *trnP* and *trnT* (gene order: 5′-*nad4L*-*trnT*-*trnP*-*nad6*-3′) was identified in all four Macrobrachium (*Macrobrachium bullatum*, *Macrobrachium lanchesteri*, *Macrobrachium nipponense* and *Macrobrachium rosenbergii*), which was similar with the out group (*Panulirus stimpsoni*^30^ & *Panulirus ornatus*^31^) in this study.

**Figure 3:**
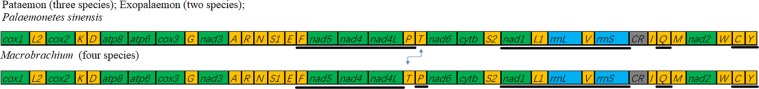
Two gene order arrangement patterns in subfamily Palaemoninae. Genes are not drawn to scale, and they are transcribed from left to right except for those indicated by underlining.

Occurrence of mitochondrial gene order rearrangement was common in Malacostraca^32–34^. Shen *et al*. identified nine different rearrangements in the comparison of 23 Pancrustacea mitogenome archived in GenBank, and found the same translocation between *E*. *carinicauda* and *M*. *rosenbergii*, which was identical to this study^10^. Wang *et al*. inferred that this invasion between *trnP* and *trnT* might be the unique mitochondrial character of genus of Exopalaemon^23^. However, from the results of present study, because the other three genera were all consistent with the same gene order pattern, Macrobrachium was supposed to be the unique genus due to its rearrangement (Figure 3).

### Phylogenetic analysis

**Figure 4:**
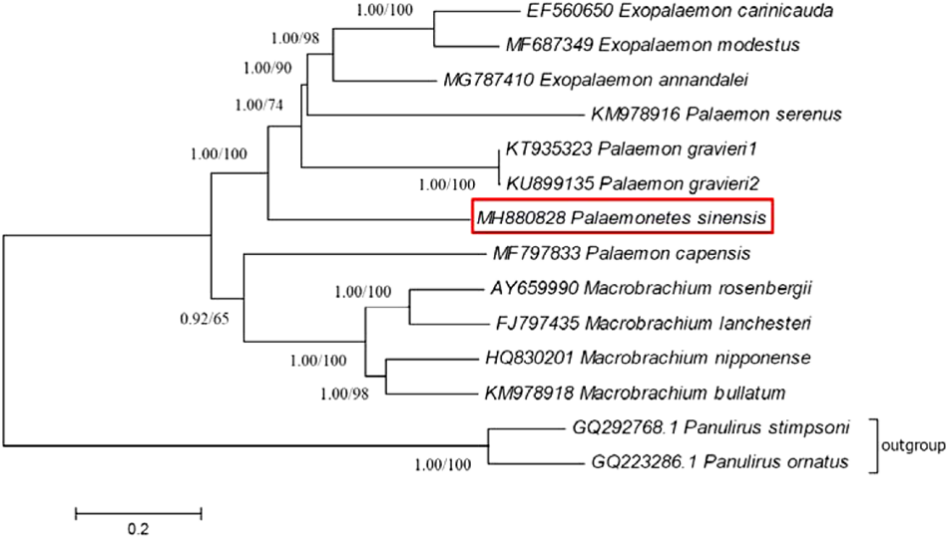
Topology derived from BI and ML of 13 concatenated mitochondrial PCGs from 14 mitogenome. Numbers beside the nodes indicate bootstrap probability of Bayesian posterior probabilities (BPP)/ML bootstrap support.

Although Palaemonidae was the second most species-rich shrimp family including 118 genera and 981 species^1^, there were only ten complete mitogenome (excluding *P*. *sinensis*) archived in GenBank so far. Phylogenetic analyses were based on the concatenated PCGs derived from 11 Palaemoninae mitogenomes belonging to four genera (Palaemonetes, Palaemon, Exopalaemon and Macrobrachium) (Table 2). As a result, same phylogenetic tree with high nodal support values for each cluster was established by both ML and BI analyses (Figure 4). Apart from the out-group, four species of Macrobrachium clustered with *P*. *capensis* in one main clade. In the other main clade, three Exopalaemon species formed a monophyletic group and then clustered with the other two Palaemon species and *P*. *sinensis* successively.

The phylogenetic relationship within subfamily Palaemoninae Rafinesque, 1815, has been always debatable in their morphological cladistics study and molecular phylogeny. Pereira demonstrated the paraphyly in Palaemon, Palaemonetes, and Macrobrachium according to the analysis a matrix of 81 morphological characters in 172 species^3^. Ashelby *et al*. strongly supported that Palaemonetes, Exopalaemon, Coutierella, and certain Palaemon belonged to single monophyletic clade based on the analyses of mitochondrial 16S rDNA and nuclear Histone (H3) genes in Palaemoninae^6^. Therefore, Ashelby *et al*. suggested a further re-appraisal of morphological characters combined with further genetic work at generic-level were needed to establish a reliable classification in Palaemoninae^6^.

In this study, apparent heterogeneity of Macrobrachium was proved by both topology and mitogenome gene order rearrange. This result supported previous study by Kim *et al*.^24^ and Shen *et al*.^10^. And also genus Exopalaemon present monophyly with high support values. Interestingly, the only species which does not distribute in Asia-Australia in this study, *P*. *capensis* merged into Macrobrachium clade with comparatively low support value. Adult *P*. *capensis* inhabit in freshwater after a more saline planktonic larval phase^35^. Its life cycle reflects the evolutionary history of freshwater palaemonid shrimp. And in this study, *P*. *sinensis*, *M*. *bullatum*, *P*. *capensis* characterized by abbreviated larval development, which has been considered as a primitive trait took place early in the origin of the family Palaemonidae^36^. The Palaemon and Palaemonetes clade confirmed their morphological similarity demonstrated by merging of species of both genera^37^, while, Cuesta *et al*.^5^ and Botello & Alvarez^38^ suggested that Palaemon and Palaemonetes were more similar, and both different from Macrobrachium according to the analysis of mitochondrial 16S rDNA. However, taking into account the tiny proportion of archived mitogenome (11 species from 3 genera in 372 species from 26 genera), more mitogenome from more complete taxon are indispensable to reveal the phylogenetic relationship within Palaemoninae.

The first complete mitogenome of genus of Palaemonates, *P*. *sinensis* was determined in this study. This result can help us to understand the basic features and gene arrange of this species. As for PCGs, in the comparison with other known mitogenomes of Palaemoninae, *P*. *sinensis* characterized by highest composition of A and C nucleotides, as well as the lowest composition of T and G nucleotides. Additionally, *P*. *sinensis* has slight positive AT-skew value and the biggest negative GC-skew value, whereas the other species all have negative AT-skew values. As for control region, *P*. *sinensis* featured by highest composition of A nucleotides, and the lowest composition of G nucleotides, as well as the highest AT-skew value. Gene order comparison of *P*. *sinensis* and previously-sequenced Palaemoninae revealed a conservative order among genera of Palaemonetes, Palaemon and Exopalaemon, and a unique translocation between *trnT* and *trnP* in Macrobrachium. The phylogenetic analysis using Bayesian Inference (BI) and Maximum Likelihood (ML) based on concatenated set of nucleotide sequences of 13 PCGs indicated that Exopalaemon formed a monophyletic group and then clustered with two Palaemon species and *P*. *sinensis* successively whereas Macrobrachium formed a monophyletic group and then clustered with *P*. *capensis* in the other clade.

## Materials and Methods

### Sample collection and DNA extraction

The *P*. *sinensis* were collected from Shenyang Longwei Lake, Liaoning, China (41°50′33.7″N; 123°35′22.3″E). The whole body of one individual shrimp was immediately preserved in liquid nitrogen until DNA extraction. Total genomic DNA was extracted using the TIANamp Marine Animals DNA Kit (TIANGEN, Beijing, China), and the quality of extracted DNA was assessed by electrophoresis on a 1% agarose gel and Thermo Scientific NanoDrop 2000.

### Genome assembly and annotation

After random break by Covairs ultrasonic breaker, DNA was fragmented for constructed genomic DNA library using Whole Genome Shotgun (WGS) strategy, which was sequenced by Illumina Miseq instrument based on NGS technology. Colinear analysis for mitochondrial splicing sequences obtained by A5-miseq v20150522^39^, SPAdesv3.9.0^40^ and BLAST v2.2.31 (https://blast.ncbi.nlm.nih.gov/Blast.cgi), were performed by using software mummer v3.1^41^ to determine the position relation of contig sequences. The complete mitogenome sequence was revised and confirmed by pilon v1.18^42^. All these procedures were performed by Shanghai Personal Biotechnology Co., Ltd., China.

The locations of putative protein-coding genes and rRNA genes were preliminarily predicted by software DOGMA^43^ and MITOS^29^, and the precise location was identified by the mitogenome of the related species based on Palaemoninae sequences archived in GenBank. Identification of initiation and termination codons were carried out by using an alignment generated through ClustalX version 2.0^44^, with other related species sequences as references, and verified by utilizing ORF finder and Blastn of NCBI. The location and secondary structure of *tRNA* genes were predicted and annotated using MITOS^29^ and tRNAscan-SE with default settings^28^. Nucleotide composition and the relative synonymous codon usage (RSCU) were determined using MEGA 7^45^.

To describe base composition, AT skew = [A − T]/[A + T], GC skew = [G − C]/[G + C] were analyzed as described by Perna & Kocher^46^. Online mitochondrial visualization tool mtviz was utilized to drawn the graphical diagram of the complete mitogenome (http://pacosy.informatik.uni-leipzig.de/mtviz/mtviz). In the end, the complete mitochondrial DNA sequence was uploaded to GenBank database under the accession number MH880828.

### Phylogenetic analysis

Eleven others complete mitogenome sequences of subfamily Palaemoninae (ten species) were obtained from GenBank (https://www.ncbi.nlm.nih.gov/genbank/) for phylogenetic analysis within Palaemoninae. GenBank sequence information of eleven species was shown in Table 2. In addition, the mitogenome of *Panulirus stimpsoni* (GQ292768.1) and *Panulirus ornatus* (GQ223286.1) were employed as an out-group taxon from GenBank. Nucleotide sequences from 13 mitogenome PCGs were aligned using Clustal Omega (https://www.ebi.ac.uk/Tools/msa/clustalo/). Moreover, Gblocks was utilized to remove poorly aligned region and divergent site^47^.

The optimal nucleotide substitution models were given by jModelTest (v2.0)^48,49^ through online server Phylemon 2 (http://phylemon.bioinfo.cipf.es/evolutionary.html) and MEGA 7^45^ based on Akaike Information Criterion (AIC) value for Maximum Likelihood method (ML) and Bayesian Information Criterion (BIC) value for Bayesian inference (BI). Consequently, GTR + I + G was selected as the best-fit evolutionary model for ML analysis by both MEGA 7^45^ and jModelTest^48,49^, whilst GTR + I + G and Tpm3uf + I + G were considered as the best model for BI analyses given by MEGA 7^45^ and jModelTest^48,49^, respectively. Because Tpm3uf model was not implemented in Mrbayes v3.2.1^50^, it was replaced by the closest over-parameterized model (GTR)^51,52^. As a result, GTR + I + G model was selected for further phylogenetic analysis.

Afterwards, ML analysis was performed on 1000 bootstrapped datasets by MEGA 7^45^. The BI analysis was carried out as 4 simultaneous Markov chain Monte Carlo (MCMC) for 100,000 generations, sampled every 100 generations by using Mrbayes v3.2.1^50^, the average standard deviation of split frequencies was less than 0.01. Both topology tree and the Bayesian posterior probilities (PP) was derived after the first 250 “burn-in” trees were excluded.

## Data Availability

The data set supporting the results of this article is available at NCBI (GenBank No. MH880828).
